# Upper Gastrointestinal Screening of Polyp Load in Children With Familial Adenomatous Polyposis: Is It Required?

**DOI:** 10.1097/PG9.0000000000000269

**Published:** 2022-12-02

**Authors:** Thomas Middleton, Ian Sugarman

**Affiliations:** From the * Sheffield Children’s Hospital; † Leeds General Infirmary.

## Abstract

**Objectives and study:**

Patients with familial adenomatous polyposis (FAP) have a propensity to form not only large bowel polyps but also upper gastrointestinal (GI) polyps with malignant potential. International guidance suggests that upper GI screening need not begin until patients are at least into their twenties. It is our experience that patients develop upper GI polyps long before this point which have the potential for malignant change.

**Methods:**

A prospective record of all upper GI endoscopies in children (aged 9 to 17) with FAP was kept across a 12-year period by 1 surgeon in our center. For each scope performed, we recorded the location, histology, and treatment of upper GI polyps.

**Results:**

Twent-eight patients aged 9 to 17 underwent a total of 48 esophagogastroduodenoscopies across a 12-year period. Thirty-eight esophagogastroduodenoscopies (79%) identified at least 1 gastric or duodenal polyp in 22 (79%) patients; 10 (36%) patients had gastric adenomas. Eight (29%) patients showed very high numbers of polyps. All 21 patients who had duodenal polyps had adenomas. None had yet developed malignancy, but 1 patient required extensive polyp excision and one is awaiting endoscopic mucosal resection.

**Conclusion:**

Our results demonstrate that young people with FAP are at the risk of developing upper GI polyps long before current guidance suggests screening the upper GI tract. We advocate for screening of the upper GI tract to start along with colonoscopy and happen at the same sitting for pediatric and young adult patients with FAP.

**What Is New**

- We found many pediatric patients with significant upper GI polyposis who would be missed under current guidelines.
- We suggest screening pediatric patients for upper GI polyps at the same time as their colonoscopies.

**What Is Known**

- Gastric and duodenal polyps are common in patients with familial adenomatous polyposis (FAP); 40% of children with FAP have duodenal adenomas when screened.
- Although not common, progress from even low-grade duodenal adenomas to duodenal cancer in under 10 years is reported.
- International guidance suggests screening need not start until patients are in their twenties.

## INTRODUCTION

Familial adenomatous polyposis (FAP) is a genetic condition whereby a fault in the adenomatous polyposis coli (APC) tumor suppressor gene leads to the formation of hundreds if not thousands of polyps in the colon. Untreated these polyps will almost inevitably lead to malignant change and death.^1^ The focus of screening in patients with—or at significant risk of—FAP, is colonoscopy. However FAP has affects beyond the colon, including the propensity to form upper gastrointestinal polyps with malignant potential.^2^ Screening for these polyps with esophagogastroduodenoscopy (OGD) is generally advocated by those who care for patients with FAP. While there is a variation in guidelines suggesting when and how often this screening should happen, in all cases, it was not until patients were adults which is justified by the fact that upper gastrointestinal (GI) cancer in children and young people with FAP is rare; an exact number is not known. The cohort reported by Bulow et al^3^ followed 368 adult patients with FAP who underwent upper GI endoscopies reporting only 6 patients with malignancy across the period with a median age of 52; however, notably the youngest patient with duodenal malignancy was 26. Others have reported that upper gastrointestinal dysplasia starts in children and young people,^4^ and it can progress to cancer in under a decade^5^; we are concerned that opportunities to pick and up and treat these polyps before they become cancerous may be missed.

In our pediatric surgical department, we regularly perform OGDs in children with FAP and have found upper GI polyps are the norm rather than the exception. We report the rates of these polyps in our patients.

## METHODS

We report the results of upper GI endoscopies done in our center for FAP. We include all OGDs performed across a 12-year period in patients with FAP by 1 pediatric surgeon up to the point they transitioned to adult care (age range 9–17). Outcomes measured were the presence of gastric and duodenal polyps, the histology of these polyps, and any treatment that was undertaken to them.

This surgeon had always performed OGDs in his FAP patients and as no deviation to patients’ care that was made from this consultant’s normal practice for this project; all data were anonymous, and no ethical approval was sought.

## RESULTS

Across a 12-year period at our center, 1 surgeon performed 48 scopes across 28 patients with an age range of 9–17 years old. Breakdown of patients is seen in Table 1.

**TABLE 1.** Patient demographics

Pt number	Age at endoscopies	Sex	Siblings screened	No. of screens	Postcolectomy and pouch?	Age at scope	Gastric polyps	Duodenal polyps	Genetics
1	12, 14, 15	M	N	3	N	12, 14, 15	Y-all	Y-1, 2	Nil
2	12, 16	F	N	2	N	12, 16	Y-both	N-both	Nil
3	15	F	Y (15)	1	Y	15	Y	N	Nil
4	13, 15	F	Y (17,18)	2	N	13, 15	Y-both	Y-both	Nil
5	11, 13, 15	M	N	3	N	11, 13, 15	Y-all	Y-3	CHRPES*
6	11, 13, 15	M	Y (19)	3	N	11, 13, 15	N-all	Y-2, 3	CHRPES* Codon 847
7	9, 11, 12, 14, 16	F	N	5	N	9, 11, 12, 14, 16	Y-all	Y-1	CHRPES Codon 847
8	13	M	N	1	N	13	Y	Y	Codon 847
9	13	M	N	1	N	13	N	N	CHRPES Codon 1213
10	15	F	Y (11)	1	N	15	N	N	Nil
11	16	M	Y (10)	1	N	16	Y	N	Nil
12	11, 13	F	N	2	N (1) Y (2)	11, 13	Y-both	Y-both	Codon 1309
13	14	F	N	1	N	14	Y	Y	Codon 1578
14	14, 16	M	N	2	N	14, 16	Y-2	N	Gardners syndrome Codon 1061
15	16	F	Y (3)	1	Y	16	Y	Y	Codon 847
16	14	M	N	1	N	14	Y	Y	Nil
17	11, 16	F	Y (4, 18)	2	N	11, 16	Y-2	Y-both	Codons 847,283
18	12, 14	F	Y (4, 17)	2	N	12, 14	N	Y-2	Codons 847,283
19	12	F	Y (6)	1	N	12	N	N	Codons 847,283
20	13	F	N	1	N	13	Y	Y	Codons 847
21	12	M	Y (23)	1	N	12	N	N	Codon 1312 + 5G>A
22	13, 14	F	N	2	N	13, 14	Y-1	N	Codon 1621 C>T
23	16	M	Y (21)	1	N	16	Y	N	Codon 1312 + 5G>A
24	12, 14, 17	M	N	3	N	12, 14, 17	Y-2, 3	N	APC deletion (including promotor region to exon 15)
25	13	M	N	2	Y	13, 14	Y-both	Y-both	Nil
26	17	M	Y (28)	1	N	17	Y	N	Nil
27	13	F	Y (26)	1	N	13	N	N	Nil
28	13	F	N	1	N	13	Y	Y	Gardners syndrome Codon 4614 C>G

Only 10 (21%) individual endoscopies (in 10 different patients) identified neither gastric nor duodenal polyps.

**FIGURE 1.**
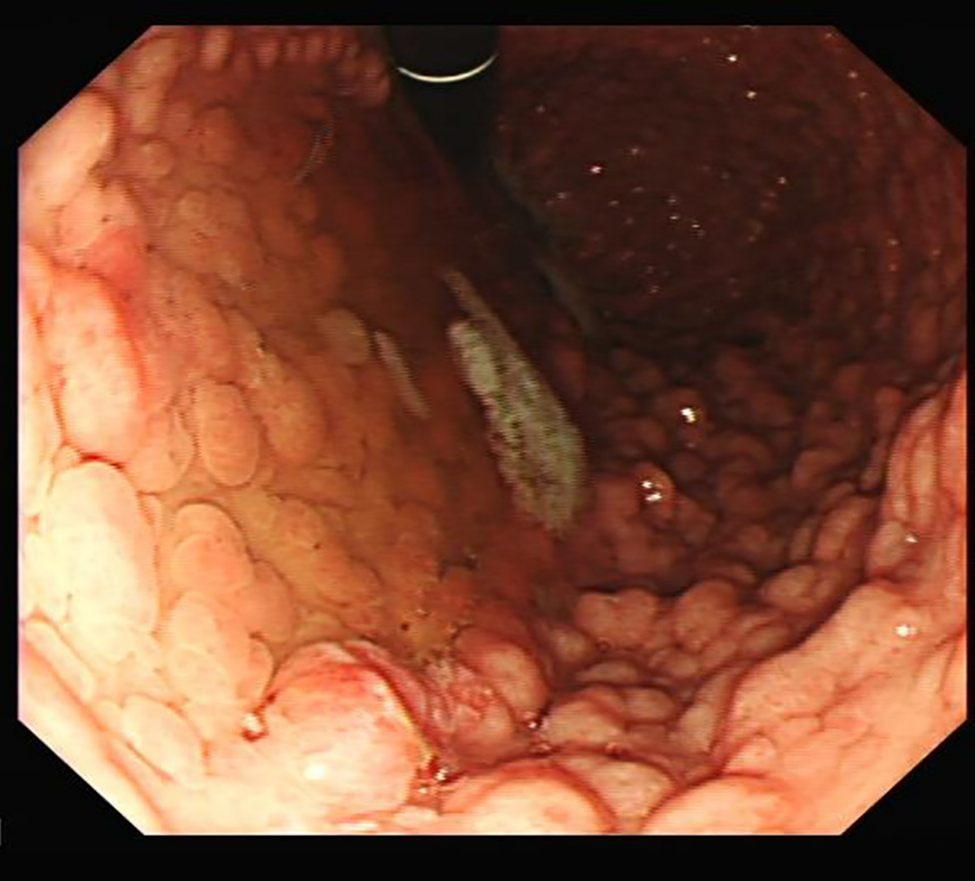
XXXXX.

Of the 21 patients with gastric polyps, 10 (35% of the total) had adenomas with or without cystic fundic polyps identified on one of their biopsies. The histology for the other children showed either normal tissue or cystic fundic polyps alone, a recognized pathology seen in FAP (see Table 2). Interestingly, 8 children were identified to have a very high gastric polyp load such as that picture shown in Figure 1. All of those children had adenomas with or without cystic fundic polyps.

**TABLE 2.** Gastric polyps

Result	No.
No. of patients	28
Total no. of OGDs	48
Total no. of scopes showing gastric polyps	36 (75%)
No. biopsied	31 (86%)
No. of scopes with very high number polyps	9 (19%)
No. of patients with very high number polyps	8 (29%)
Histology	
Cystic	18 (59% of biopsied)
Adenomatous	6 (19% of biopsied)
Mixed	6 (19% of biopsied)
Normal	1 (3% of biopsied)
Treatment	
Cold biopsy	27 (75%)
None	9 (25%)

Of the 19 (68%) children who had pathological duodenal polyps, the histology was always an adenoma (Table 3).

**TABLE 3.** Duodenal polyps

Result	No.
No. of patients	28
Total no. of OGDs	48
Total no. of scopes showing duodenal polyps	21 (44%)
No. biopsied	21 (100%)
Histology	
Adenomatous	19 (90%)
Normal	2 (10%)
Treatment	
Cold biopsy	16 (76%)
Argon laser (and planned endomucosal resection)	1 (5%)
None	4 (19%)

While the majority of the children had a Spigelman score of 1 or 2, one child has a Spigelman score of 3, had undergone endomucosal resection of polyps and is having regular surveillance (Supplemental Digital Content Figure 1; http://links.lww.com/PG9/A97).

With the records kept, one further piece of information we have tried to record is that of where the codon abnormality is in the APC gene. Table 1 summarizses this finding.

## DISCUSSION

Upper GI polyps, particularly duodenal polyps, are extremely common in FAP with rates of duodenal polyposis over 90% by 70 years of age.^3^ Furthermore duodenal and ampullary carcinoma are the most common cause of death from FAP in patients post total colectomy^6^ with a relative risks as high as 330% above the general population observed in previous studies.^7^

The degree of risk polyps can be scored by giving the patient a Spigelman score (see Supplemental Digital Content Table 2; http://links.lww.com/PG9/A97) according to number of polyps, polyp size, type of histology, and degree of dysplasia.

The time taken for polyps to progress to adenocarcinoma is variable. Saurin et al^8^ followed up 35 patients for an average of 4 years and found that half of them increased their Spigelman score.

There is a host of different guidance advising on the screening for upper GI polyps in FAP, the majority suggesting of which was in adult life. The American Society of Colon and Rectal Surgeons guidance^9^ is for screening to start between the ages of 20 and 25 and then space screening according to the Spigelman grade at endoscopy. The American College of Gastroenterologists^10^ suggest upper GI screening to start later still at 25 to 30 years of age (and again space screening according to Spigelman grade).

The UK guidance from The British Society of Gastroenterology and The Association of Colproctology for Great Britain and Ireland is for upper GI screening to start at the age of 25 and continue every 3 years for those with confirmed FAP and “if/when a clinical diagnosis of FAP is made based on colorectal phenotype” in those without a genetic diagnosis, but with clinical suspicion.^11^

Different guidance again is advocated by the European Society of Medical Oncology^12^ who advocate upper GI screening to start when the patient age is 25–30 and to happen every 5 years.

To summarize, all advocated screening starts from age 20 and over, and screening happens every 1 to 5 years thereafter. Patients as young as 9 years of age in our group had both gastric and duodenal polyps which following any of these sets of guidance would have not been picked up over a decade.

Although this is a large number of pediatric patients reviewed, in terms of all patients with FAP, it is relatively a small group. The importance of this article is that one child in this group had a Spiegelman score of 3 and one must be concerned that had he not been picked up at this age, he may have presented as an adult with a potential duodenal cancer. Sourrouille et al,^13^ in their article, concluded that >20% of patients develop high-grade dysplasia with duodenal polyposis after 10 years. We therefore question whether the current guidelines for the age for commencement of screening of the upper GI tract in FAP is correct.

We recognize the limitations of our study as being relatively small and limited to the experience of 1 surgeon in 1 center. We also have not shown any children progressing to come to actual harm. The evidence shows that polyps become significantly dysplastic in just a few years. We have not, however, recorded Spigelman grading for all the patients we screened for and we have not found evidence to prove that dysplasia in upper GI polyps progresses quickly children with FAP, although it would be logical to expect this to be the case.

In similar findings to ourselves, Gutierrez Sanchez et al retrospectively reviewed all the OGDs done in pediatric patients with FAP at their center (the Mayo Clinic) and went on to do a meta-analysis of the literature on this subject.^4^ They found the rate of duodenal adenomas at 52% in their own cohort at first OGD and 40% in the literature review including one case report of a 12-year-old with high-grade duodenal dysplasia.^14^

Clinically significant duodenal dysplasia in pediatric patients with FAP is rare, but it does exist, and the finding of Spigelman grade 3 disease in a 14-year-old in our cohort is an evidence of this. We suggest there may be a role for OGD at the time of the first colonoscopy for pediatric patients with FAP. OGDs are generally very safe procedures and the risk to the child who is already undergoing a general anesthetic for a colonoscopy is minimal; the additional cost in terms of money, time, and resource use should also be relatively small. Undertaking such an approach to screening the upper GI tract in children with FAP may allow polyps to be picked up much earlier and may prevent the potential malignancy they may lead to.

## Supplementary Material


