# Supplementary material for: Upper Gastrointestinal Screening of Polyp Load in Children With Familial Adenomatous Polyposis: Is It Required?
Source: JPGN Rep. 2022 Dec 2;4(1):e269. doi: 10.1097/PG9.0000000000000269 (PMC10754608; doi:10.1097/PG9.0000000000000269)
Supplement: Supplementary file 1 [file pg9-4-e269-s001.pdf]

Supplementary online figure 1

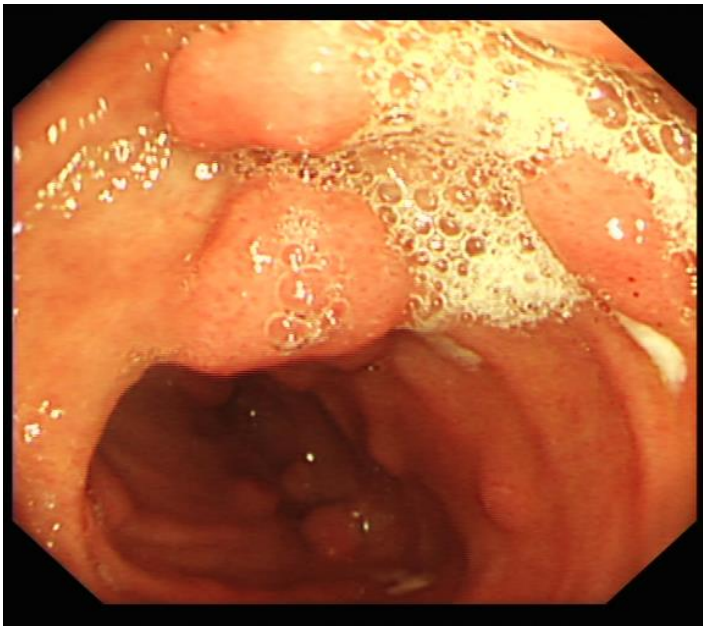

Supplementary online table 1- genetic anomalies

Gene abnormality	No of patients	Number with high polyp load
Deletion exons 7-15	1	1
Codon 847	8	2
Codon 1213	1	0
Codon 1309	1	0
Codon 1061	1	0
Codon 1312+5G>A	2	0
Codon 1621 C>T	1	1
Codon 1032	1	0
Codon 4616 C>G	1	1

Supplementary online table 2- Spigellman classification

Variables	1 point	2 points	3 points
Number of polyps	1-4	5-20	>20
Polyp size (mm)	1-4	5-10	>10
Histology	Tubular	Tubovilloua	Villous
Dysplasia	Mild	Moderate	Severe
